# CPT1A-Mediated Fatty Acid Oxidation Promotes Precursor Osteoclast Fusion in Rheumatoid Arthritis

**DOI:** 10.3389/fimmu.2022.838664

**Published:** 2022-02-22

**Authors:** Zhaoyang Huang, Rong Luo, Liu Yang, Haiqi Chen, Xinyao Zhang, Jiawen Han, Hongxia Wang, Zhongyang Zhou, Zhao Wang, Lan Shao

**Affiliations:** ^1^ The Center for Translational Medicine, The First Affiliated Hospital, Sun Yat-Sen University, Guangzhou, China; ^2^ Department of Rheumatology, The Third Affiliated Hospital, Guangzhou Medical University, Guangzhou, China; ^3^ Department of Urology, The First Affiliated Hospital, Sun Yat-Sen University, Guangzhou, China; ^4^ Laboratory Medicine Center, Nanfang Hospital, Southern Medical University, Guangzhou, China; ^5^ Department of Orthopaedics, The Third Affiliated Hospital, Guangzhou Medical University, Guangzhou, China

**Keywords:** CPT1A, fatty acid oxidation, osteoclast fusion, fusogenic molecule, clathrin, rheumatoid arthritis

## Abstract

The overproduction of osteoclasts, leading to bone destruction in patients with rheumatoid arthritis (RA), is well established. However, little is known about the metabolic dysfunction of osteoclast precursors (OCPs) in RA. Herein, we show that increasing fatty acid oxidation (FAO) induces OCP fusion. Carnitine palmitoyltransferase IA (CPT1A), which is important for carnitine transportation and is involved in FAO in the mitochondria, is upregulated in RA patients. This metabolic change further increases the expression of clathrin heavy chain (CLTC) and clathrin light chain A (CLTA) by enhancing the binding of the transcription factor CCAAT/enhancer binding protein β (C/EBPβ) to the promoters of *CLTA* and *CLTC*. This drives clathrin-dependent endocytosis pathway, which attenuates fusion receptors in the cellular membrane and contributes to increased podosome structure formation. This study reveals a new mechanism through which FAO metabolism participates in joint destruction in RA and provides a novel therapeutic direction for the development of drugs against bone destruction in patients with RA.

## Introduction

Rheumatoid arthritis (RA) is a systemic autoimmune disease characterized by progressive destruction of joint cartilage and bone (1). Osteoclasts are multinucleated cells that play a critical role in the pathological arthritic bone erosion in patients with RA (2). They can be formed *via* cell–cell fusion of circulating mononuclear precursor cells, which are derived from pluripotent hematopoietic stem cells. Osteoclast precursors (OCPs) exhibit a monocyte phenotype, upon stimulation by macrophage colony-stimulating factor (M-CSF) and receptor activator of nuclear factor-κB ligand (RANKL) (3, 4). Recent studies also suggested that arthritis-associated OCPs in the pannus originate from circulating blood monocytes but not from locally resident macrophages. Peripheral blood monocytes isolated from patients with RA have enhanced osteoclast genesis capability compared with that from healthy controls (HCs) (5, 6), suggesting that circulating OCPs (monocytes) play a vital role in bone erosion in RA.

During osteoclast differentiation, mononucleated OCPs undergo cell–cell fusion, leading to the formation of multinucleated osteoclasts (7, 8). A direct relationship between osteoclast size and resorption activity was also observed (9). Dendritic cell-specific transmembrane protein (DC-STAMP), vacuolar (H^+^) ATPase (v-ATPase), metalloproteinase domain-containing protein (ADAM) 8, ADAM12, CD9, CD44, CD47, CD36, and osteoclast stimulatory transmembrane protein (OC-STAMP) are involved in osteoclast fusion and multinucleation (7, 10–13). Furthermore, the depletion of osteoclast fusogenic molecules such as CD-9, DC-STAMP, and OC-STAMP attenuated the formation of multinuclear osteoclasts, which led to the inhibition of OC resorption (14, 15). This suggests that fusogenic molecule expression and their cellular membrane distribution in OCPs may play a critical role in giant multinucleated osteoclastogenesis in RA patients.

Fatty acids β-oxidation (FAO) is the process by which fatty acids are converted to products, such as acetyl-CoA, to generate energy. To enter the mitochondria for oxidation, the fatty acids are first conjugated to free carnitine by carnitine palmitoyltransferase I (CPT1), which is the rate-limiting enzyme in long- and medium-chain FAOs. The main subtypes of CPT1 in monocytes are CPT1A and CPT1B. This carnitine–fatty acid complex is then shuttled through the mitochondrial membrane and releases the fatty acid acyl-CoA groups from carnitine with the help of carnitine palmitoyltransferase II (CPT2) (16, 17). Importantly, in the context of the RA environment, monocytes are more susceptible to using FAO to supply energy (18, 19). The key components of the FAO metabolic pathway such as cholesterol and fatty acid metabolites have a functional impact on RA monocytes and drive RANKL-induced osteoclastogenesis (6, 20–23). However, how FAO energy metabolic pathway influences OCP fusion during osteogenesis in patients with RA remains poorly understood.

Herein, we report that the circulating OCPs in RA patients upregulate the CPT1A-mediated FAO metabolic pathway. In addition, we show that enhanced FAO not only influences osteoclastogenesis, but also promotes cell–cell fusion during osteoclast maturation. The knockdown of the *CPT1A* gene and the use of a CPT1A activity inhibitor blocked OCP fusion and osteoclastogenesis. Mechanistic studies indicated that the activity of CPT1A upregulated clathrin expression by facilitating CCAAT/enhancer binding protein β (C/EBPβ) binding to the promoters of clathrin light chain A (*CLTA*) and clathrin heavy chain (*CLTC*). Therefore, the clathrin-dependent endocytosis pathway, which drives fusogenic molecule accumulation in the cellular membrane and podosome structure formation, was activated. These results indicate that the activation of the CPT1A mediated FAO metabolic pathways in circulating OCPs is associated with giant multinuclear osteoclast formation in patients with RA.

## Material and Methods

### Patients and Control Individuals

The study group included 71 individuals with a diagnosis of RA and 69 healthy controls (HCs). The RA patients fulfilled the American College of Rheumatology (ACR) criteria (24) or the ACR/European League Against Rheumatism (EULAR) 2010 classification criteria for RA (25), and all RA patients were positive for rheumatoid factor and/or anticyclic citrullinated peptide antibody. The control subjects were matched with age, gender and ethnicity. A history of cancer, uncontrolled medical disease or any other inflammatory syndrome were excluded. Healthy individuals didn’t have any personal or family history of autoimmune disease. The demographic characteristics of the RA patients and HC donors are summarized in **Table 1**. The study was approved by the Medical Ethics Committee of Hospital and all subjects provided appropriate informed consent.

**Table 1:** Demographic and clinical characteristics of the study populations.

Characteristics	HC	OA	RA	*P*
**Demographic**				
Number of subjects	69	8	71	
Female/Male^a^	47 /22	6 /2	52 /19	0.78
Age (mean±SD years)^a^	50.7±4.2	53.7±2.4	51.9±3.1	0.29
**Disease statu**s				
Disease duration (mean±SD years)		N/A	7.5±1.8	
Active disease		N/A	86.1 %	
Tobacco use		N/A	15.2%	
Extra-articular manifestations		N/A	43.5%	
ESR, mm/h		N/A	37.9±3.1	
DAS28-CRP (mean±SD)		N/A	3.6±0.4	
DMARD naïve		N/A	11.1%	
**Radiographic status**				
Total modified Sharp score (mean±SD)			24.7±19.4	
Joint erosion subscore (mean±SD)			17.2±18.6	
**Medications**				
Corticosteroids		N/A	70.8%	
Methotrexate		N/A	66.7%	
Hydroxychloroquine		N/A	56.9%	
Leflunomide		N/A	25.0%	
TNF-α inhibitors		N/A	12.5%	

a No significant difference, RA patients compared with HC donors.

ESR, erythrocyte sedimentation rate; DAS28, Disease Activity Score in 28 joints; DMARD, disease-modifying antirheumatic drugs; an active disease defined by Food and Drug Administration (FDA) criteria [presence of three or more of the following: morning stiffness (> 45 min), swollen joints (> 3), tender joints (> 6) and sedimentation rate (> 20 mm/h)]. N/A, Not Applicable.

RA disease activity was assessed using the Disease Activity Score in 28 joints with 4 variables, including C-reactive protein level. Radiographs of bilateral hands, wrists, and feet (anteroposterior view) were performed on all RA patients and assessed with the modified Sharp/van der Heijde score. Erosive disease was defined according to the 2013 EULAR definition when a cortical break was detected by radiography (6, 26).

### Cell Purification and Culture

Human Peripheral blood mononuclear cells (PBMCs) were collected using Ficoll-Paque™ PLUS (Mediatech, Inc., Herndon, VA, USA) gradient centrifugation. To sort CD14^+^ cells, PBMCs were positively selected with CD14 microbeads using autoMACS (Miltenyi Biotec Inc., Auburn, CA, USA).

### Osteoclast Differentiation and Bone Resorption Assay

For osteoclast differentiation, 1×10^5^ purified CD14^+^ monocytes were seeded in 96-well plates or 2 × 10^5^ in a 24-well plate (Corning, USA) per well. Change the fresh medium in the presence of 100 ng/ml RANKL and 50 ng/ml M-CSF every four days, finally terminate the differentiation in 21st day. Then the cells were stained by Tartrate Resistant Acid Phosphatase Staining (TRAP) staining kit and FITC-labeled phalloidin (Merck, Darmstadt, Germany). Cells including at least 3 nuclei and bands of F-actin containing podosomes were defined as mature osteoclasts, and finally mature osteoclasts were counted including the number of per well, the nuclei of each mature osteoclast, and the sizes of the osteoclasts were obtained by measuring the diameters of multinucleated TRAP-positive cells using Olympus IX71 inverted wide-field fluorescence microscope.

For bone resorption assay, bovine cortical bone slices were layered at the bottom of 96-well culture plates, and 1 × 10^5^ peripheral blood CD14^+^ monocytes were seeded onto the slices and cultured in the presence of 100 ng/ml of recombinant human RANKL and 50 ng/ml of M-CSF. Resorption pits on the slices were shown by toluidine blue staining and measured using an ImageJ 1.47 analysis system (NIH).

To test for the role of FAO in mediating osteogenesis and cell–cell fusion, etomoxir (MedChemExpress, Monmouth Junction, NJ, USA), which inhibiting the CPT1A enzyme activity results in a reduction in fatty acid oxidation, were used at concentrations of 25 μM. Etomoxir was added to each exchange of the osteoclast differentiation medium.

### RNA Isolation and Quantitative Real-Time PCR

Total RNA was extracted from 1 × 10^5^ cells, and cDNA was synthesized with the AMV-reverse transcriptase and random hexamer primers (Roche Diagnostic Corp., Indianapolis, USA). Reverse transcription was performed using a standard procedure (Super Script First-Strand Synthesis System; Invitrogen) using 1 μg of total RNA. QPCR was performed using the iQ SYBR Green Supermix on the iCycler Real-Time Detection system (Bio-Rad, Hercules, CA, USA) on an StepOne Plus Real-Time Detection system (Applied Biosystems, USA) (27). The gene expression was normalized to GAPDH and the relative amount of mRNA was calculated using the 2^−ΔΔCt^ method. Primer sequences were listed in **Table 2**. The relative amount of mRNA was normalized with a housekeeping *GAPDH* as indicated and was quantified.

**Table 2:** Primer sequences for qPCR.

Target gene	Sense primer (5’-3’)	Antisense primer (5’-3’)
FSP27	CAAGACTAGGAACCCTGAAGCC	CCCTTCTGGAGGACCATGAAC
PLIN1	CCCAGGAGTGACAGGAATTGTT	TGAGGCCTTTGTTGACTGCC
FASN	AGAACTTGCAGGAGTTCTGG GACA	TCCGAAGAAGGAGGCATCAAA CCT
SCD	ATTGGGTGGCTGTTTGTTCG	ACCACAAAGCACATGAGCAC
ACADS	ATCGCCATGGAGGAGATCAG	GAACCCATGAGTCGCCCTC
ACADM	GGGTTCGGGCGATGCTG	ACCAAGTTCCCAGGCTCTTC
ACADL	CTTTGCAACACCCAGTACGC	AGCCTTTCCTGTGGAAGCTC
ACADVL	CAAGACTAGGAACCCTGAAGCC	GCCAGCTTGGGGAGGTATTT
HADHB	TTCCCACTGCATCAAAATGGG	ATGCAACAAACCCGTAAGCG
ACOX1	GATATCGCCATTCCCCAGAG	ATGGCACTTTTCCTGACAGC
CPT1A	TTCAGTTCACGGTCACTCCG	TGACCACGTTCTTCGTCTGG
CPT1B	CCTGGTGCTCAAGTCATGGT	CGGTCCAGTTTACGGCGATA
RUNX2	GGTGTTCCAAAGACTCCGGC	TACGCATCACAACAGCCACA
TRAP	CGTATTCTCTGACCGCTCCC	TCTTGAAGTGCAGGCGGTAG
Cathepsin K	CTGGCTATGAACCACCTGGG	AAGGGTGTCATTACTGCGGG
MMP9	AGTCCACCCTTGTGCTCTTC	CCCGAGTGTAACCATAGCGG
NFATc1	GTGGCAGCCATCAACGCCCT	TACGAGGCCTGTGGCACCGA
ADAM8	ACACTGTCGTCATTCCATGC	AAGGACGTAGCTCACCCTCT
ATP6V0D2	AGCTGTTTCACCCTACCTTGG	CTTGCATCCTCGAACCAGGC
CD47	ACACTGTCGTCATTCCATGC	TCAGTGGGGACAGTGGACTT
CD36	TGTCATTGGTGCTGTCCTGG	CTCAGCGTCCTGGGTTACAT
DC-STAMP	GGCCCTTGTGGTTGGAAGTA	CACAGGGCCTCTGTTGATGT
CTLA	AAAGCAAACAACAGGGCAGC	GGAGACATCTTTGGCCTGCT
CTLC	GCACTGAAAGCTGGGAAAACT	CTGCAAGGCTAGAATGGCGA
C/EBPβ	CACAGCGACGACTGCAAGATCC	CTTGAACAAGTTCCGCAGGGTG

### Immunoblotting

Whole cell lysates were prepared in RIPA buffer (Cell signaling Technology Danvers, MA, USA) plus phenylmethylsulphonyl fluoride (PMSF) and a protease inhibitor cocktail (Merck, Germany) (28). For each sample, equal amounts of total protein were electrophoresed, transferred to a nitrocellulose membrane and blocked with 5% non-fat milk. Antibodies specific for CPT1A, CPT1B, CLTA, CLTC, C/EBPβ, and Actin (Santa Cruz Biotechnology, Santa Cruz, CA, USA) were added 12 h at 4°C, followed by washing with washing buffer. Membranes were subsequently incubated with secondary antibodies (1:5000; Santa Cruz) for 1 h at room temperature and developed with a chemiluminescent detection system (Beyotime, Shanghai, China).

### Flow Cytometry

For cell fusion analysis, CD14^+^ monocytes were pretreated with 100 ng/ml of RANKL and 50 ng/ml of M-CSF for 3 days. Then cells were surface stained with APC-CD14, FITC-CD36, FITC-DC-STAMP, and FITC-CD47 mouse polyclonal antibodies (BioLegend, San Diego, CA, USA).

### Immunofluorescence Staining

Peripheral blood CD14^+^ monocytes were plated on 24-well culture plates with coverslips. After incubation, the medium was aspirated and cells were washed twice in PBS, fixed in 4% paraformaldehyde for 15-30 minutes, permeabilized in 0.2% Triton X-100 for 10 minutes at room temperature for the exposure of intracellular antigen, and blocked in PBS containing 3% bovine serum albumin (BSA; Affymetrix) for 30 minutes. Cells were stained with a mouse anti-human CD14 monoclonal antibody, rabbit anti-human DC-STAMP, rabbit anti-human CD36, mouse anti-human clathrin, and FITC-labeled phalloidin overnight at 4°C. Alexa Fluor 594–conjugated goat anti-rabbit IgG and Alexa Fluor 488–conjugated goat anti-mouse IgG (1:1,000; concentrations of stock solutions 2 mg/ml) (life science) were added and incubated for 1 hour at 37°C. After washing in PBS, the nucleus was stained with 4’6-diamindino-2-phenylindole (DAPI, Sigma-Aldrich) and coverslips were mounted with ProLong^®^ Gold Antifade Reagent (Life Technologies). The images were analyzed using a Zeiss LSM 880 Confocal Imaging System (Zeiss, Jena, Germany) (29).

### RNA Interference

To knockdown *CPT1A* expression, monocytes were pretreated with 100 ng/ml of RANKL and 50 ng/ml of M‐CSF for 4 days, 6 ug siRNA *CPT1A* and control vector were transfected into cells by oligofectamine (Life Technologies, Inc.; Invitrogen), following protocols provided by the manufacturer. The sequences of CPT1A siRNA were 5′-GCACCGUCAAUGCCUACAA dTdT-3′. For osteoclast differentiation, peripheral blood CD14^+^ monocytes were transfected with *CPT1A* sequence–specific short hairpin RNA (shRNA) expression lentivirus (pLV-mCherry-U6>*CPT1A*_shRNA) (VectorBuilder, Guangzhou, China) with (pLV-mCherry-U6>Scramble_shRNA) as a control. Stably transduced cells were selected using puromycin.

### Chromatin Immunoprecipitation

The ChIP assay was performed using ChIP kit (Merck, Germany) with C/EBPβ antibody (Santa Cruz Biotechnology, USA) (30). Cells in a 10-cm culture plate were crosslinked with 1% formaldehyde for 10 minutes. Crosslinking was neutralized with 0.2M glycine. Cells were collected and suspended in lysis buffer. Genomic DNA was released by using SDS Lysis Buffer, and sonicated to 200-1000 bps. Protein–DNA complexes were precipitated with C/EBPβ antibody (Cell Signaling Technology, USA). After purification, the precipitated DNA and input were de-crosslinked at 65°C and then purified. The capacity of C/EBPβ binding to *CLTA* and *CLTC* promoter were quantified by qPCR. Primer sequences used in ChIP-qPCR were as follows: *CLTA* sense 5’-ATGGCCCAGATGGAGAAAGC-3’ and antisense 5’-GGGAGGTGTTGGATGTGAGG-3’; *CLTC* sense 5’-ATGGCCCAGATGGAGAAAGC-3’ and antisense 5’-TGTTTCGACTGAGCCCCT-3’.

### Lipidomics Analysis

The liquid chromatography method was established on the QExactive LC (Thermo Fisher, Waltham, MA, USA) system, where Ultimate 3000 LC (Thermo Fisher) was used for the liquid phase portion. The mass spectrometry method was established in QExactive (Thermo Fisher) system. The lipidomics mass spectrometry method was performed in full scan/data-depend ms^2^ Top10 analysis mode, and positive/negative spectrum analysis in ESI mode. Spray voltage was +3500V/-3000V, the sheath gas was 40arb, the auxiliary gas was 10arb, the ion transfer tube temperature was 320°C, the auxiliary gas heating temperature was 350°C. The first-level full scan resolution was 70000, and the mass range was 100-1500m/z. Ms^2^ had a secondary resolution of 17500, top 10, impact energy of 20, 40, and mass range of 70-1500m/z.

The data obtained from the above lipidomics method were imported into Lipid Search 4.1 software for Lipid identification and queue analysis. The full scan/data-depend ms^2^ data collected by QExactive instrument was selected to use the daughter ion mode for lipid identification. The accurate mass deviation threshold of parent ion and daughter ion was set to 5ppm, and isotope matching was performed to display the ions with fragment matching score above 2. The retention time window threshold was set to 1min. Positive mode selects +H peak, +Na peak, +NH4 peak addition and negative mode selects -H peak, +HCOO peak addition and sum for database retrieval. The retrieval results select and identify the ions with grade A, B, and C for queue analysis to obtain the lipid profile information and the relevant data results could be exported.

### Statistical Analysis

Data were analyzed using SPSS version 13.0 software. Groups were compared using unpaired Student’s t-test as appropriate. The results were expressed as the mean ± standard error of the mean (SEM). The correlation of parametric data was assessed using Pearson’s correlation test. P values of less than 0.05 were considered significant.

## Results

### Decreased Lipid Accumulation in the Patients With RA

**Figure 1:**
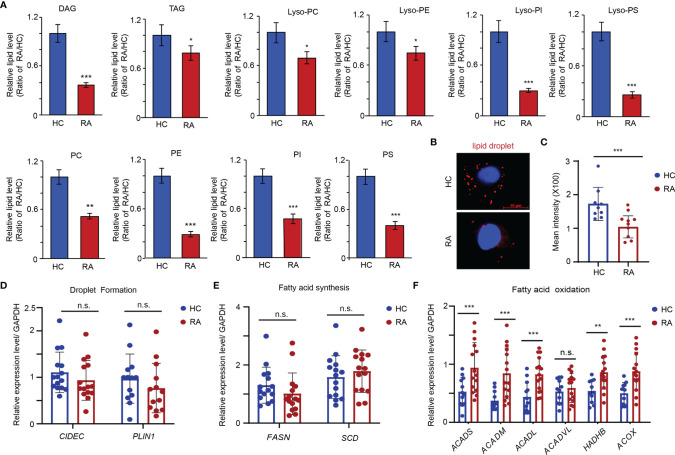
Enhanced Fatty acid oxidation in the precursor of osteoclasts of RA patients. CD14^+^ monocytes were isolated from RA patients and HCs. **(A)** Metabolite analysis of lipid including diacylglycerol (DAG), triacylglycerols (TAG), phosphatidylcholine (PC), phophatidylethanolamine (PE), phosphatidylnositol (PI), phosphatidylserine (PS), lyso-PC, lyso-PE, lyso-PI and lyso-PS content in the monocytes from HC (n = 3) and RA patients (n = 3). The results shown as the ratio of lipid levels of RA/lipid levels of HC. **(B, C)** Neutral lipid droplet were stained with Nile red and quantified with software of image J at **(C)**. **(D)** Transcription level of *FSP27* and *PLIN1* expression was quantified by qPCR in monocytes from 15 RA patients and 15 HCs. The results from 3 independent experiments. **(E)** Transcription level of *FASN* and *SCD5* expression was quantified by qPCR in monocytes from 15 RA patients and 15 HCs. The results from 3 independent experiments. **(F)** Transcription level of *ACADS*, *ACADM*, *ACADL*, *ACADVL*, *HADHB*, and *ACOX* were quantified by qPCR in monocytes from 15 RA patients and 15 HCs. The results from 3 independent experiments. All data was presented as the mean ± SEM. *P < 0.05; **P < 0.01; ***P < 0.001; n.s. no significance.

Lipids are major molecular constituents of cells that have important functions in membrane structure, energy production, and signal transduction. To study alterations in the lipid metabolic pathway of OCPs in RA, the lipidome of peripheral CD14^+^ monocytes isolated from RA patients and HCs was comprehensively examined by liquid chromatography coupled with dynamic quantitative mass spectrometry. Overall, the lipid profiles of RA patients contained markedly reduced amounts of diacylglycerol (DAG), triacylglycerol (TAG), phosphatidylcholine (PC), phophatidylethanolamine (PE), phosphatidylnositol (PI), phosphatidylserine (PS), lyso ***-*** PC, lyso ***-*** PE, lyso ***-*** PI, and lyso ***-*** PS than those of HCs (**Figure 1A**). To further characterize the metabolic state of lipids in RA monocytes, CD14^+^ monocytes from RA patients and age-matched HCs were subjected to Nile red staining to examine the accumulation of intracellular lipid droplets. Deletion of cytoplasmic neutral lipids in RA-monocytes resulted in a red intensity that was 1.74-fold lower in RA monocytes than in those from HCs, suggesting either impaired lipid synthesis or enhanced FAO in RA monocytes (**Figures 1B, C**).

Next, the gene expression of *CIDEC* (*FSP27*) and *PLIN1*, which is critical for the process of droplet formation, was examined (31). Expression of both genes was comparable to that of HCs (**Figure 1D**). Similarly, no significant differences in the key genes involved in lipid biosynthesis (*FASN* and *SCD*) were observed (**Figure 1E**) (31). However, the genes that are relevant for mitochondrial FAO pathway (*ACADS*, *ACADM*, *ACADL*, *HADHB*, and *ACOX*) were expressed at significantly higher levels in RA monocytes than HCs (**Figure 1F**), suggesting the reduced content of lipids in RA monocytes induced by metabolic activation of the FAO pathway.

### Elevated CPT1A Expression in CD14^+^ Monocytes From RA Enhances OCP Fusion

**Figure 2:**
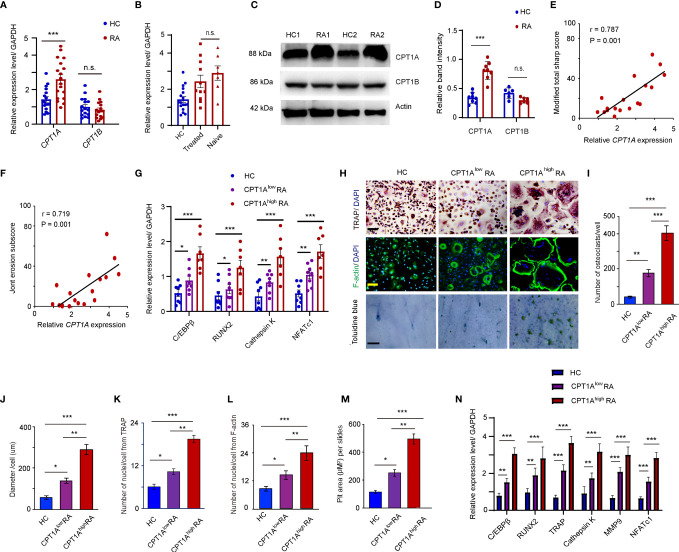
Enhanced CPT1A expression in OCPs promotes osteoclast formation and cell-cell fusion. CD14^+^ monocytes were isolated from HCs and RA patients. **(A)** *CPT1A* and *CPT1B* gene expression was quantified by qPCR in monocytes from HCs and RA patients. The results from 3 independent experiments involving 18 RA patients and 17 HCs are shown as scatter plots. **(B)** Higher expression of *CPT1A* is independent of drug treatment (treated vs. treatment naïve). **(C–D)** Representative blotting for CPT1A and CPT1B protein expression. The band intensities were adjusted to actin. Results analyzing 5 RA patients and 5 HCs are shown as the mean ± SEM at **(D)**. **(E, F)** *CPT1A* gene expression in 18 RA patients was assessed for correlation with the modified total Sharp score **(E)**, and erosion subscore **(F)**. **(G–M)** CD14^+^ monocytes from 17 healthy controls, 11 patients with lower *CPT1A* gene expression, and 7 patients with higher *CPT1A* gene expression were cultured with RANKL and macrophage colony-stimulating factor (M-CSF). **(G)** Transcript levels of *C/EBPβ*, *RUNX2*, *Cathepsin K*, and *NFATc1* at day 0 were quantified by qPCR in HC, CPT1A^low^ RA, and CPT1A^high^ RA groups. **(H)** Mature osteoclasts were detected by tartrate-resistant acid phosphatase (TRAP) staining on day 21, bar 100 μm; and FITC–phalloidin staining on day 21, bar 100 μm. Bone resorption activity of osteoclasts was detected by toluidine blue staining on day 21. bar 50 μm. Representative results are shown. **(I)** Cell counts of mature osteoclasts from 24-well plates on day 21. **(J)** The sizes of the osteoclasts were obtained by measuring the diameters of multinucleated TRAP-positive cells. **(K)** Mean nuclei number of multinucleated TRAP-positive cells. **(L)** Mean nuclei number of multinucleated F-actin positive cells. **(M)** Pit area of bone resorption lacunae. **(N)** Transcript levels of *C/EBPβ*, *RUNX2*, *TRAP*, *Cathepsin K*, *MMP9*, and *NFATc1* were quantified by qPCR in HC, CPT1A^low^ RA, and CPT1A^high^ RA groups. All data was presented as the mean ± SEM. *P < 0.05; **P < 0.01; ***P < 0.001; n.s. no significance.

CPT1 is the rate-limiting enzyme for β-oxidation of long- and medium-chain lipids in the mitochondria (16). To assess which subtype of CPT1 is upregulated in the CD14^+^ monocytes of patients with RA, the mRNA levels of *CPT1A* and *CPT1B* were determined in the CD14^+^ monocytes from age-matched HCs and RA patients. A significantly higher expression of *CPT1A*, but not *CPT1B*, was observed in the CD14^+^ monocytes of patients with RA than in those from the HCs (*CPT1A*, HCs:1.430 ± 0.081 vs. RA: 2.594 ± 0.112, *P* < 0.001; *CPT1B*, HCs: 0.949 ± 0.081 vs. RA: 0.878 ± 0.059, *P* = n.s.) (**Figure 2A**). To assess the impact of immunosuppressive therapy and the disease state on *CPT1A* induction, *CPT1A* expression was examined in patients with newly diagnosed RA who were not treated with disease-modifying anti-rheumatic drugs or underwent corticosteroid treatment within 3 months before recruitment to the study. There was no significant difference between *CPT1A* levels in the treated and untreated patients (**Figure 2B**), suggesting that drugs are not the causative factors of CPT1A upregulation. The increased expression of CPT1A protein, but not CPT1B in RA patients was also validated by western blotting (**Figures 2C, D**). Furthermore, there was a positive correlation observed between *CPT1A* gene expression in peripheral blood CD14^+^ monocytes and total modified Sharp/van der Heijde score (r = 0.787, P = 0.001) (**Figure 2E**) and erosion subscore (r = 0.719, P = 0.001) (**Figure 2F**), suggesting that high CPT1A expression in circulating osteoclast precursors may be associated with bone erosion in RA.

To explore a possible connection between CPT1A expression and osteoclastogenesis, the RA patients were further divided into two groups based on the relative gene expression levels of *CPT1A* in CD14^+^ monocytes, *CPT1A* ^low^ group (*CPT1A* < 3) and *CPT1A* ^high^ group (*CPT1A* > 3). The gene expression levels of *C/EBPβ*, *RUNX2*, *Cathepsin K*, and *NFATc1*, which associated with osteoclast-prone phenotype and function, were compared in the fresh isolated CD14^+^ monocytes from HC, RA patients with lower expression level of *CPT1A*, and RA patients with higher *CPT1A* expression. The strongly enhanced expression levels of those genes were observed in *CPT1A* ^high^ group than those in CPT1A ^low^ and HC groups (**Figure 2G**). This result indicated that *CPT1A* expression may associated with the dysfunction of osteoclast differentiation in RA patients. The samples were then cultured in the presence of 100 ng/ml RANKL and 50 ng/ml M-CSF for 21 days. TRAP staining and FITC-labeled phalloidin were used to detect mature osteoclasts in 24-well plates. The results in **Figure 2H** show that RA monocytes have a greater ability to form osteoclasts, as the numbers of mature osteoclasts in both groups of RA monocytes were much higher than that in HCs (HCs: 53.4 ± 3.8; *CPT1A* ^low^ RA: 155.6 ± 19.6; *CPT1A* ^high^ RA: 387.6 ± 46.7) (**Figure 2I**). As the overall size of the osteoclasts and number of nuclei were positively correlated with their resorption capability (32), our results also indicate that osteoclasts in the CPT1A^low^ RA and *CPT1A* ^high^ RA samples have a larger diameter (HCs: 47.20 ± 5.6 μm; CPT1A^low^ RA: 79.60 ± 9.7 μm; CPT1A^high^ RA 203.0 ± 18.2 μm), more nuclei for TRAP and F-actin (TRAP, HCs: 6.1 ± 0.7, CPT1A^low^ RA: 10.9 ± 1.2, CPT1A^high^ RA: 18.9 ± 1.7; F-actin, HCs: 9.2 ± 1.5, CPT1A^low^ RA: 16.1 ± 2.4, CPT1A^high^ RA: 22.3 ± 2.6) staining and significant higher pit area of bone resorption lacunae than those in HCs (HCs: 144.5 ± 15.7; CPT1A^low^ RA: 223.2 ± 14.9; CPT1A^high^ RA: 433.8 ± 22.6) (**Figure 2J**–**M**). In particular, the number of mature osteoclasts was significantly higher in RA patients with higher expression levels of *CPT1A* compared to patients with lower *CPT1A* expression (*P* <0.01), as was also found in case of osteoclast diameter (*P* <0.01), number of nuclei per osteoclast (*P* <0.01), and the pit area of bone resorption lacunae on day 21 (*P* <0.01) (**Figure 2I**–**M**). Meanwhile, induction of the genes associated with osteoclast differentiation and activity, such as *C/EBPβ*, *RUNX2*, *Cathepsin K*, *TRAP*, *MMP-9* and *NFATc1* was also detected using qPCR. There was significantly higher expression of these genes in the monocytes isolated from RA patients with higher CPT1A than those of patients with lower CPT1A and HCs (**Figure 2N**). This suggests that upregulation of CPT1A expression is not only associated with osteoclastogenesis but also with OCP fusion, which contributes to the formation of giant osteoclasts in RA patients.

### Elevated Cell–Cell Fusion Molecular Expression in RA Monocytes

**Figure 3:**
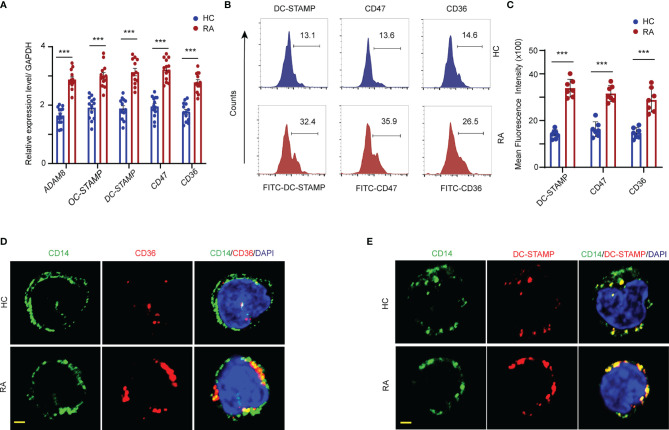
FAO promotes fusogenic molecular expression in the cellular membrane. CD14^+^ monocytes were isolated from RA patients and controls. **(A)** Transcript levels of *ADAM8*, *OC-STAMP*, *DC-STAMP*, *CD47*, and *CD36* were quantified by qPCR from RA patients and HCs. The results from 3 independent experiments. **(B, C)** The DC-STAMP, CD47, CD36 membrane expression level in monocytes were analyzed by flow cytometry. Representative histograms for one HC (blue) and one RA (red); three independent experiments examining 6 HCs and 7 RA patients are presented as the mean ± SEM. **(D, E)** Localization of DC-STAMP and CD36 in the cellular membrane of monocytes were detected by immunofluorescence staining. Bar 10 μm. All data shown as the mean ± SEM. ***P < 0.001.

Osteoclast fusion and multinucleation are pivotal steps in osteoclastogenesis. To assess osteoclast fusion dynamics under RA inflammatory conditions, the mRNA expression of *ADAM8*, *OC-STAMP*, *DC-STAMP*, *CD47*, and *CD36* in the CD14^+^ monocytes from RA patients and HCs was detected by qPCR. The abundance of *ADAM8*, *OC-STAMP*, *DC-STAMP*, *CD47*, and *CD36* mRNA was significantly higher in patients with RA than that in the HCs (**Figure 3A**). In addition, flow cytometry confirmed an approximately two-fold increase in in the mean fluorescence intensity (MFI) of DC-STAMP, CD47, and CD36 in the OCPs from RA patients compared to those in the HCs (**Figures 3B, C**).

To further assess the cellular membrane accumulation of the fusogenic molecules, DC-STAMP and CD36 expression was visualized by co-immunostaining with CD14, which is a cellular surface marker of monocytes. Elevated DC-STAMP/CD14 and CD36/CD14 co-localization signals, shown in yellow, were observed on the cell surface of monocytes in patients with RA. In contrast, the expression levels of both DC-STAMP and CD36 in the surface membranes were markedly lower in the monocytes of HCs than in those of RA patients (**Figures 3D, E**).

### FAO Inhibition or CPT1A Silencing Rescued Osteoclastogenesis and Fusion

**Figure 4:**
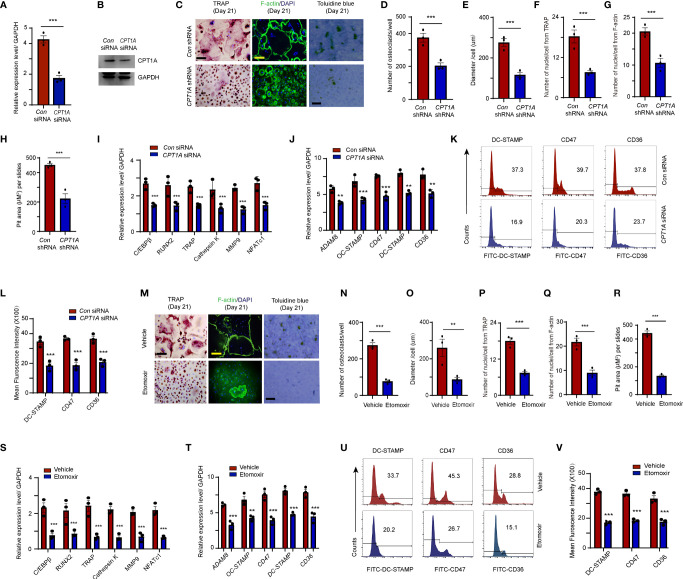
Fusogenic molecule membrane accumulation in OCPs is regulated by CPT1A. **(A–K)** CD14^+^ monocytes isolated from RA patients were transfected with control and *CPT1A*-specific siRNA or *CPT1A shRNA* after M-CSF and RANKL stimulation at day 2. **(A, B)** *CPT1A* siRNA. **(A)** *CPT1A* transcript levels were quantified by qPCR 24 h after control and *CPT1A* siRNA transfection. **(B)** Representative blotting for CPT1A expression. **(C–G)** Osteoclast differentiation. CD14^+^ monocytes from RA patients were transfected with short hairpin RNA expression lentivirus for *CPT1A* knockdown (pLV-mCherry-U6>*CPT1A*_shRNA) or with (pLV-mCherry-U6 >Scramble_shRNA) as a control. **(C)** Mature osteoclasts were detected by TRAP, bar 100 μm; and FITC–phalloidin staining on day 21. bar 100 μm. Bone resorption activity of osteoclasts was detected by toluidine blue staining on day 21. bar 50 μm**. (D)** Cell counts of mature osteoclasts from 24-well plates. **(E)** Mean diameter of mature osteoclasts. **(F)** Mean nuclei number of multinucleated TRAP-positive cells. **(G)** Mean nuclei number of multinucleated F-actin positive cells. **(H)** Pit area of bone resorption lacunae. Results from 3 RA patients are shown. **(I)** Transcript levels of *C/EBPβ*, *RUNX2*, *TRAP*, *Cathepsin K*, *MMP9*, and *NFATc1* were quantified by qPCR. **(J)** Transcript levels of fusogenic molecule expression were quantified by qPCR. **(K)** DC-STAMP, CD47, and CD36 membrane expression were analyzed by flow cytometry. Representative histograms for one control siRNA (blue) and one *CPT1A* siRNA (red); three independent experiments from 3 RA are shown at **(L)**. **(M–V)** CPT1A inhibitor. RA-derived monocytes were treated with control or 25 μM etomoxir after M-CSF and RANKL stimulation. **(M–R)** Mature osteoclasts were detected by TRAP, bar 100 μm; and FITC–phalloidin staining on day 21, bar 100 μm. Bone resorption activity of osteoclasts was detected by toluidine blue staining on day 21. bar 50 μm. **(N)** Cell counts of mature osteoclasts from 24-well plates. **(O)** Mean diameter of mature osteoclast. **(P)** Mean nuclei number of multinucleated TRAP-positive cells. **(Q)** Mean nuclei number of multinucleated F-actin positive cells. **(R)** Pit area of bone resorption lacunae. Results from 3 RA patients are shown. **(S)** Transcript levels of *C/EBPβ*, *RUNX2*, *TRAP*, *Cathepsin K*, *MMP9*, and *NFATc1* were quantified by qPCR. **(T**) Transcript levels of fusogenic molecule were quantified by qPCR. **(U)** The DC-STAMP, CD47, and CD36 membrane expression in monocytes were analyzed by flow cytometry Representative histograms for one control (blue) and one etomoxir (red); three independent experiments examining 3 RA are shown at **(V)**. All data was presented as the mean ± SEM. **P < 0.01; ***P < 0.001.

To assess whether elevated CPT1A levels contribute to osteoclastogenesis and fusion of circulating OCPs in RA, CD14^+^ monocytes derived from RA patients were transfected with small interfering RNA or lentiviral vector with specific *CPT1A* sequence to knock down the *CPT1A* expression. The transfected cells were cultured with 100 ng/ml RANKL and 50 ng/ml M-CSF for 21 days. *CPT1A* sequence-specific knockdown markedly reduced CPT1A gene and protein expression (**Figures 4A, B** and **Supplementary Figure 1A**), and also decreased the numbers of mature osteoclasts (*con* shRNA: 389.8 ± 17.2 vs. *CPT1A* shRNA: 202.47 ± 31.1, *P* < 0.001), cell diameter (*con* shRNA: 271.4 ± 23.6 μm vs. *CPT1A* shRNA: 102.1 ± 15.6 μm, *P* < 0.001), nuclei per osteoclast (TRAP, *con* shRNA: 18.4 ± 2.1 vs. *CPT1A* shRNA: 7.8 ± 0.9, *P* < 0.001; F-actin, *con* shRNA: 20.1 ± 2.8 vs. *CPT1A* shRNA: 8.4 ± 1.0, *P* < 0.001), and pit area of bone resorption lacunae (*con* shRNA: 453.4 ± 11.5 vs. *CPT1A* shRNA: 222.6 ± 22.5, *P* < 0.001) (**Figures 4C**–**H**). In addition, *CPT1A* knockdown resulted in the decrease in the expression of osteoclastogenesis genes (*C/EBPβ*, *RUNX2*, *Cathepsin K*, *TRAP*, *MMP9*, and *NFATc1)* (**Figure 4I**) and fusogenic genes that participated in OCP fusion (*ADAM8*, *OC-STAMP*, *DC-STAMP*, *CD47*, and *CD36*) (**Figure 4J**). This result also was conformed through the flow cytometry analysis of the cellular membrane expression of DC-STAMP, CD47, and CD36 (**Figures 4K, L**).

Alternatively, RA-derived CD14^+^ monocytes were treated with etomoxir, a pharmacological inhibitor that specifically inhibits the CPT1A-mediated FAO metabolic pathway. We did not find any distinguishable difference in the transcript levels of *CPT1A* after etomoxir treatment compared to those in the vehicle group, which is consistent with the previous studies demonstrated that etomoxir binds irreversibly to the catalytic site of CPT-1 and etomoxir treatment does not change the mRNA level of *CPT1* in rat tissues (33, 34). However, this inhibitor effectively suppressed osteoclastogenesis-associated gene expression (*C/EBPβ*, *Cathepsin K*, and *NFATc1*). Combining with the results from *CPT1A* gene specific knockdown which rule-out the possible off-target effects of the etomoxir, it may suggest that this inhibitor mainly work through the inhibition of CPT1A enzymatic function (**Supplementary Figure 1B**). Furthermore, treatment with etomoxir resulted in a significant decrease in the cell number (Vehicle: 277.4 ± 26.8 vs. Etomoxir: 76.7 ± 13.1, *P* < 0.001), size (Vehicle: 257.6 ± 57.2 μm vs. Etomoxir: 92.47 ± 15.3 μm, *P* < 0.01) and the number of nuclei per osteoclast (TRAP, Vehicle: 18.1 ± 2.0 vs. Etomoxir: 8.2 ± 1.1, *P* < 0.001; F-actin, Vehicle: 22.8 ± 2.7 vs. Etomoxir: 9.3 ± 1.9, *P* < 0.001) of mature osteoclasts, as well as and pit area of bone resorption lacunae in patients with RA (Vehicle: 437.2 ± 11.7 vs. Etomoxir: 142.13 ± 9.9, *P* < 0.001) (**Figures 4M-R**).

Consistently, reduced mRNA expression osteoclastogenesis genes and cellular membrane distribution of fusogenic molecules were found in the monocytes treated with etomoxir (**Figures 4S**–**V**). In summary, these results confirm that CPT1A directly regulates OCP fusion.

### CPT1A Upregulation of OCP Fusion Through the Increase of Clathrin-Dependent Endocytosis

**Figure 5:**
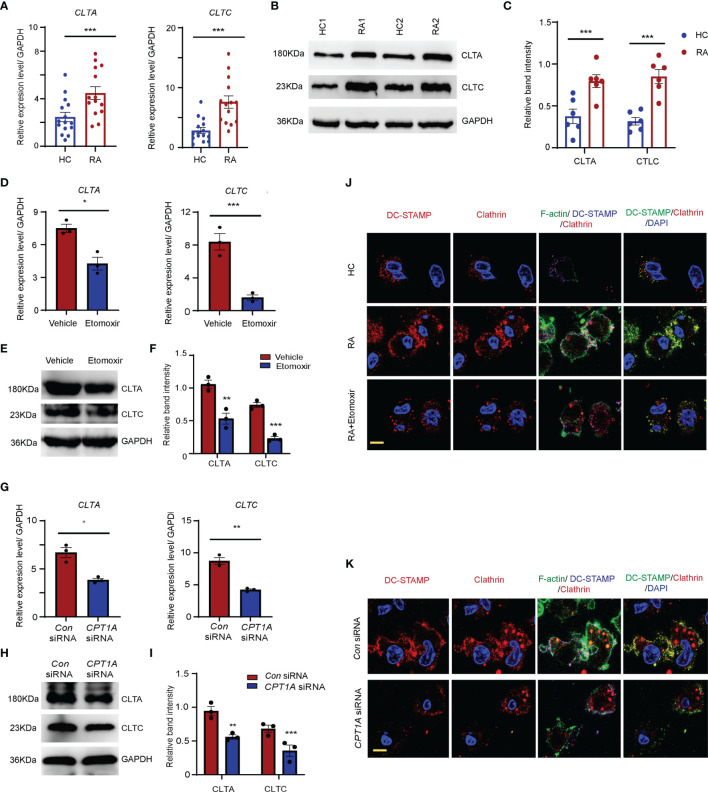
CPT1A promotes OCP fusion through upregulating clathrin dominant endocytosis. CD14^+^ monocytes were isolated from HCs and RA patients. **(A)** Clathrin light chain A (*CLTA*) and clathrin heavy chain (*CLTC*) expression was quantified by qPCR in monocytes from RA patients and HCs. The results from 3 independent experiments involving 14 RA patients and 15 HCs are shown. **(B, C)** Representative blotting for CLTA and CLTC protein expression. The band intensities were adjusted to GAPDH. The western blotting experiment results analyzing 8 RA patients and 8 HCs are shown as the mean ± SEM at **(C)**. **(D–F)** CD14^+^ monocytes were treated with DMSO or 25 μM etomoxir at day 2 after M-CSF and RANKL stimulation. **(D)** *CLTA* and *CLTC* gene expression was quantified by qPCR. The results from 3 independent experiments. **(E, F)** Representative blotting for CLTA and CLTC. The band intensities were adjusted to GAPDH. The western blotting experiment results analyzing 3 RA patients are shown at **(F)**. **(G–I)** CD14^+^ monocytes were transfected with control or *CPT1A*-specific siRNA after M-CSF and RANKL stimulation at day 2. **(G)** *CLTA* and *CLTC* gene expression was quantified by qPCR. The results from 3 independent experiments. **(H, I)** Representative blotting for CLTA and CLTC. The band intensities were adjusted to GAPDH. The western blotting experiment results analyzing 3 RA patients are shown at **(I)**. **(J, K)** Monocytes were labelled with FITC–phalloidin staining (green) and DC-STAMP antibody (blue). Podosome structures were determined with co-staining with clathrin (red) antibody in HC, RA, and RA treated with 25 μM etomoxir **(J)**; or in RA patients transfected with control or *CPT1A*-specific siRNA **(K)**. Representative images are from one of three HC and RA patients. Bar, 10 μm. Data are representative from three independent experiments is shown. All data shown as the mean ± SEM. * *P* < 0.05; **P < 0.01; ***P < 0.001.

Clathrin-mediated endocytosis plays a critical role in the cellular surface distribution of fusogenic molecules and OCP fusion (10). To evaluate whether clathrin-dominant endocytosis is augmented OCPs that are in the peripheral blood of RA patients, CD14^+^ monocytes were purified from PBMCs of both RA patients and HCs. There was a significant increase in the transcription levels of *CLTA* and *CLTC* in the monocytes of patients with RA compared to those in the HCs (*CLTA*: HCs: 2.5 ± 0.3 vs. RA: 4.7 ± 0.6, *P* < 0.001; *CLTC*: HCs: 2.8 ± 0.2 vs. RA: 7.6 ± 0.4, *P* < 0.001) (**Figure 5A**). Western blotting results also showed that both the CLTA and CLTC band intensities were significantly upregulated in RA-derived monocytes compared to those in HCs (**Figures 5B, C**).

To clarify whether clathrin expression is regulated by the CPT1A-mediated FAO metabolic pathway, CD14^+^ monocytes purified from RA patients were treated with etomoxir or *CPT1A* specific RNA interference (**Figures 5D**–**F**). Impaired CPT1A enzymatic activity and *CPT1A* gene expression significantly downregulated the mRNA and protein levels of CLTA and CLTC in the monocytes from RA patients (**Figures 5G**–**I**).

Podosomes or podosome-like structures are required for OCP fusion (35, 36). Next, the effect of clathrin upregulation on OCP fusion in RA was assessed. To do this, the formation of podosome structure was examined in RA patients and HCs at day 3 after the induction of osteoclast differentiation. In RA patients, markedly elongated F-actin–rich protruding structure were found between OCPs, which had clear DC-STAMP and clathrin co-localization with the F-actin ring. The number of podosome structures was significantly higher in the OCPs of RA patients than those of the HCs (**Figures 5J, K**). Furthermore, upon RANKL stimulation, DC-STAMP was internalized by clathrin-dependent mechanisms in RA OCPs with DC-STAMP co-localization with clathrin endocytic vesicles. However, this colocalization signal was much less in HCs. Both the protruding F-actin-rich structures and colocalization of DC-STAMP and clathrin endocytic vesicles were successfully disrupted by CPT1A enzymatic inhibition or *CPT1A* knockdown (**Figures 5J, K**), indicating that CPT1A promotes OCP fusion in RA patients *via* activating clathrin-dependent endocytic activity.

### FAO Enhances C/EBPβ Binding to CLTA and CLTC Promoters

**Figure 6:**
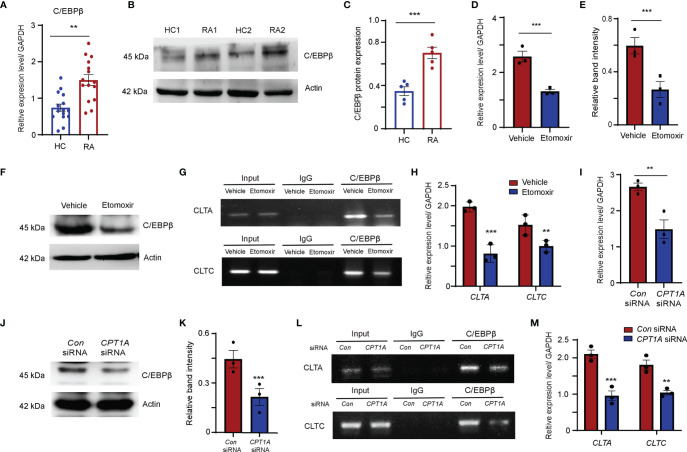
FAO pathway promotes clathrin expression through enhanced transcript factor C/EBPβ binding to the promoters of *CLTA and CLTC*. **(A–C)** CD14^+^ monocytes were isolated from HC and RA patients and stimulated with M-CSF and RANKL. **(A)** *C/EBPβ* gene expression was quantified by qPCR. The results from 3 independent experiments. **(B)** Representative blotting for C/EBPβ expression. The band intensities were adjusted to actin. The western blotting experiment results analyzing 6 HCs and 6 RA patients are shown at **(C)**. **(D–H)** CD14^+^ monocytes were isolated from RA patients were treated with DMSO or 25 μM etomoxir at day 2 after M-CSF and RANKL stimulation. **(D)** *C/EBPβ* gene expression was quantified by qPCR. The results from 3 independent experiments. **(E, F)** Representative blotting for C/EBPβ expression. The western blotting experiment results analyzing 3 RA patients are shown. **(G, H)** Chromatin immunoprecipitation (ChIP) assay. **(G)** ChIP assays with C/EBPβ antibody were performed in the monocytes isolated from 3 RA patients and treated with DMSO or 25 μM etomoxir. **(H)** Bindings of C/EBPβ to the *CLTA* and *CLTC* promoters were quantified by qPCR. The results from 3 independent experiments. **(I–M)** CD14^+^ monocytes were isolated from RA patients were transfected with control or *CPT1A*-specific siRNA after M-CSF and RANKL stimulation at day 2. **(I)** *C/EBPβ* gene expression was quantified by qPCR. The results from 3 independent experiments. **(J, K)** Representative blotting for C/EBPβ expression. The western blotting experiment results analyzing 3 RA patients are shown at **(K)**. **(L, M)** Chromatin immunoprecipitation (ChIP) assay. **(L)** ChIP assays with C/EBPβ antibody were performed in the monocytes isolated from 3 RA patients transfected with control or *CPT1A*-specific siRNA. **(M)** Bindings of C/EBPβ to the *CLTA* and *CLTC* promoters were quantified by qPCR. The results from 3 independent experiments. All data are mean ± SEM. **P < 0.01; ***P < 0.001.

C/EBPβ is a master transcription factor involved in lipid metabolism and also involved in osteoclast differentiation (37–39). The results of transcript factor analysis also suggested that C/EBPβ is a potential transcription factor may bind to the promoters of *CLTA* and *CLTC*. Therefore, C/EBPβ expression in the monocytes was examined in patients with RA and HCs. The abundance of C/EBPβ mRNA and protein in the monocytes from RA patients was significantly higher than in those of HCs (**Figures 6A**–**C**). To clarify whether enhanced C/EBPβ expression is regulated by CPT1A, the transcript and protein expression levels of C/EBPβ were detected in RA monocytes treated with or without etomoxir. Both transcript and protein expression levels of C/EBPβ were dramatically reduced in the RA monocytes cultured with etomoxir (**Figures 6D**–**F**).

To further investigate whether C/EBPβ could bind to the promoters of *CLTA* and *CLTC*, C/EBPβ chromatin immunoprecipitation (ChIP) assays was performed in RA-derived monocytes treated with or without etomoxir. ChIP analysis results confirmed the immunoprecipitation of C/EBPβ and the *CLTA* and *CLTC* promoters (**Figure 6G**). Clear bands that decrease C/EBPβ bindings to the *CLTA* and *CLTC* promoters in RA monocytes supplied with etomoxir were observed (**Figures 6G, H**).

In an alternative approach, the function of CPT1A was inhibited by siRNA interference by transfecting RA monocytes with *CPT1A* specific-interfering sequences. Reduction in *CPT1A* gene expression significantly downregulated the transcript and protein expression levels of C/EBPβ (**Figures 6I**–**K**), and also strongly attenuated the binding of C/EBPβ to the *CLTA* and *CLTC* promoter regions, indicating that CPT1A-mediated FAO metabolism upregulates *CLTA* and *CLTC* expression by promoting C/EBPβ binding to the *CLTA* and *CLTC* promoters (**Figures 6L, M**).

## Discussion

The present study revealed that in comparison to those from HCs, osteoclasts derived from RA patients have a larger size and more nuclei, which are formed by the fusion of OCPs during osteogenesis. The CPT1A-mediated FAO metabolic pathway is highly activated in CD14^+^ monocyte found in the peripheral blood of RA patients. The activated FAO metabolic pathway increased the expression of the C/EBPβ transcription factor and enhanced its binding to the promoters of *CLTA* and *CLTC* in peripheral OCPs from RA patients. This led to an increase in the expression of clathrin, which triggered the clathrin-dependent endocytosis pathway and resulted in the increased distribution of fusogenic molecules on the cellular surface. Following this, the fusogenic molecules facilitated the formation of fusion-specific podosomes in the OCPs of RA.

Osteoclastogenesis is an energy-consuming process that is achieved through FAO metabolism and glycolysis (40). A study using the Seahorse Extracellular Flux Analyzer revealed that monocyte metabolism is more susceptible to FAO, which rapidly eliminates lipid droplet storage in cells (41). It was observed that both the lipid content and the frequency of lipid droplets were significantly lower in the RA-derived monocytes as a result of the activation of β-oxidative metabolism. CPT1A, an isoform of CPT1, plays a critical role in the upregulation of FAO in the monocytes taken from patients with RA (16, 42, 43). A clear increase in the expression of CPT1A in CD14^+^ monocytes was observed in patients with RA, which was positively correlated with the radiographic scores. Elevated CPT1A levels in RA monocytes promoted osteoclast differentiation and giant multinuclear osteoclast formation. These results imply that increased expression of CPT1A in circulating monocytes may be involved in enhanced OCP fusion in patients with RA.

Asynchronous fusion of OCPs within the monocyte–macrophage lineage is a key step in osteoclast formation and efficient bone resorption (4). Studies have shown that multinucleated giant osteoclasts attained through monocyte fusion have increased bone resorption compared to that of other mononuclear osteoclasts (9). Specifically, osteoclast fusogenic molecules, such as DC-STAMP, ADAM8, OC-STAMP, CD47, and CD36, that traffic between the cell membrane and plasma drive this process (11). The surface distribution of those fusogenic molecules increased in the OCP before osteoclast fusion, and rapidly declines after the completion of fusion (14, 44–46). Indeed, a significantly higher expression of those fusogenic molecules was found in the surface membrane of OCPs in patients with RA, indicating that cellular membrane accumulation of fusogenic molecules enhances OCP fusion in RA. Etomoxir, an inhibitor of CPT1A, effectively attenuated the cellular membrane distribution of these molecules and other osteoclastogenesis gene expression. However, the suppression of FAO by etomoxir is subtle, as it has diverse effects on fatty acid metabolism and energy production (33, 34, 47). Moreover, we did not find change in the transcript levels of *CPT1A* upon etomoxir treatment. Similar results were reported in previous studies in etomoxir-treated rats (33, 34). The issue is whether etomoxir decreases osteoclast differentiation through inhibition of CPT1A enzymatic function or other side effects which are not specific for CPT1A. To exclude the possible off-target effects of the etomoxir, *CPT1A* gene knockdown with lentiviral construct of *CPT1A* shRNA and *CPT1A*-specific siRNA were performed to verify the effects of CPT1A on osteoclast differentiation and fusogenic molecule expression. Downregulation of *CPT1A* expression markedly reduced both the osteoclastogenesis gene expression and cellular membrane distribution of these fusogenic molecules. These results implying that the increased expression of fusogenic proteins in the cellular membrane is directly regulated by CPT1A in patients with RA.

In particular, osteoclast fusion requires the formation of podosome protrusions (10), which are highly dynamic actin-rich structures that are involved in multiple cell functions (48–50). Clathrin-coated endocytic activity combined with enriched DC-STAMP expression was observed on the cellular protrusions formed from adjacent cells. As the cellular surface distribution of fusogenic molecular such as DC-STAMP is required for the formation of these structures (10), it suggests that clathrin-dependent endocytosis may lead to cellular surface accumulation of fusogenic molecules. Increased amount of podosome structures with attached clathrin and DC-STAMP proteins were clearly observed in the OCPs of RA patients surrounding the sites of OCP–OCP fusion, which is much less in those of HCs. Consistently, these structures were disrupted by CPT1A gene knockdown and the use of CPT1A pharmacological agents, indicating that activated clathrin-mediated endocytosis and podosome structure formation is dependent on CPT1A regulated FAO metabolic pathway.

C/EBPβ senses lipid status in cells and augments lipid accumulation with the aid of FAO suppression (51). However, whether FAO regulates C/EBPβ expression remains unclear. This study showed that, a feedback loop between fatty acid metabolism and C/EBPβ expression was present in the monocytes of patients with RA. The overactivated FAO pathway also increased C/EBPβ expression to enhance fatty acid uptake. Importantly, C/EBPβ plays a critical role in regulating clathrin expression. C/EBPβ binds to the promoters of *CLTA* and *CLTC*, resulting in improved expression of these genes and aggravated OCP fusion. Inhibition of CPT1A activity in RA monocytes reduces C/EBPβ binding to the promoters of *CLTA* and *CLTC*. Our results clearly show that, in RA monocytes, activated FAO enhanced C/EBPβ binding to the promoters of *CLTA* and *CLTC*, promoting their transcription and subsequently enhancing clathrin-mediated endocytosis.

## Conclusion

Our study identified a new mechanism of the CPT1A-mediated FAO metabolic pathway, which promotes OCP fusion by upregulating fusogenic molecular cellular membrane accumulation *via* enhancing clathrin-dependent endocytosis. The present findings indicate that target CPT1A and fatty acid metabolism in peripheral CD14^+^ monocytes could be a therapeutic strategy to inhibit OCP multinucleation, which may be a promising therapeutic target for bone loss in patients with RA.

## Data Availability Statement

The original contributions presented in the study are included in the article/**Supplementary Material**, futher inquiries can be directed to the corresponding author/s.

## Ethics Statement

The studies involving human participants were reviewed and approved by the Medical Ethics Committee of the First Affiliated Hospital, Sun Yat-Sen university. The patients/participants provided their written informed consent to participate in this study.

## Author Contributions

All authors were involved in drafting the article or revising it critically for important intellectual content. Study conception and design, LS, ZH, RL, and LY. Acquisition of data, RL, ZH, LY, HC, XZ, JH, HW, ZZ, and ZW. Analysis and interpretation of data, ZH, RL, LY, and LS.

## Funding

This work was supported by the National Natural Science Foundation of China (grant no. 82071818), the Guangdong Natural Science Foundation (grant no. 2021A1515011030).

## Conflict of Interest

The authors declare that the research was conducted in the absence of any commercial or financial relationships that could be construed as a potential conflict of interest.

## Publisher’s Note

All claims expressed in this article are solely those of the authors and do not necessarily represent those of their affiliated organizations, or those of the publisher, the editors and the reviewers. Any product that may be evaluated in this article, or claim that may be made by its manufacturer, is not guaranteed or endorsed by the publisher.

## Supplementary Material

Supplementary Figure 1 mRNA levels of *CPT1A* and osteoclast differentiation markers at different stages of OC development. **(A)** *CPT1A* shRNA. CD14^+^ monocytes from RA patients were transfected with short hairpin RNA expression lentivirus for *CPT1A* knockdown (pLV-mCherry-U6>*CPT1A*_shRNA) or with (pLV-mCherry-U6>Scramble_ shRNA) as a control. Transcript levels of *CPT1A*, *C/EBPβ*, *Cathepsin K*, and *NFATc1* were quantified by qPCR on day 5, day 12, and day 18 during osteoclast differentiation. **(B)** CPT1A inhibitor. RA-derived monocytes were treated with control or 25 μM etomoxir after M-CSF and RANKL stimulation. Transcript levels of *CPT1A*, *C/EBPβ*, *Cathepsin K*, and *NFATc1* were quantified by qPCR on day 5, day 12, and day 18 during osteoclast differentiation.

## Abbreviations

M-CSF, Macrophage Colony-Stimulating Factor; RANKL, Receptor Activator Of Nuclear Factor-κB Ligand; DC-STAMP, Dendritic Cell-Specific Transmembrane Protein; OC-STAMP, Osteoclast Stimulatory Transmembrane Protein; v-ATPase, vacuolar (H^+^) ATPase; ADAM 8, A Disintegrin And Metalloproteinase Domain 8; FAO, Fatty Acids β-oxidation; CPT1, Carnitine Palmitoyltransferase I; CPT2, Carnitine Palmitoyltransferase II; OCP, Osteoclast Precursor; PBMCs, Peripheral Blood Mononuclear Cells; TRAP, Tartrate Resistant Acid Phosphatase Staining; C/EBPβ, CCAAT Enhancer Binding Protein β; CLTA, Clathrin Light Chain A; CLTC, Clathrin Heavy Chain.
