# Supplementary figures and images for: CPT1A-Mediated Fatty Acid Oxidation Promotes Precursor Osteoclast Fusion in Rheumatoid Arthritis
Source: Front Immunol. 2022 Feb 22;13:838664. doi: 10.3389/fimmu.2022.838664 (PMC8902079; doi:10.3389/fimmu.2022.838664)

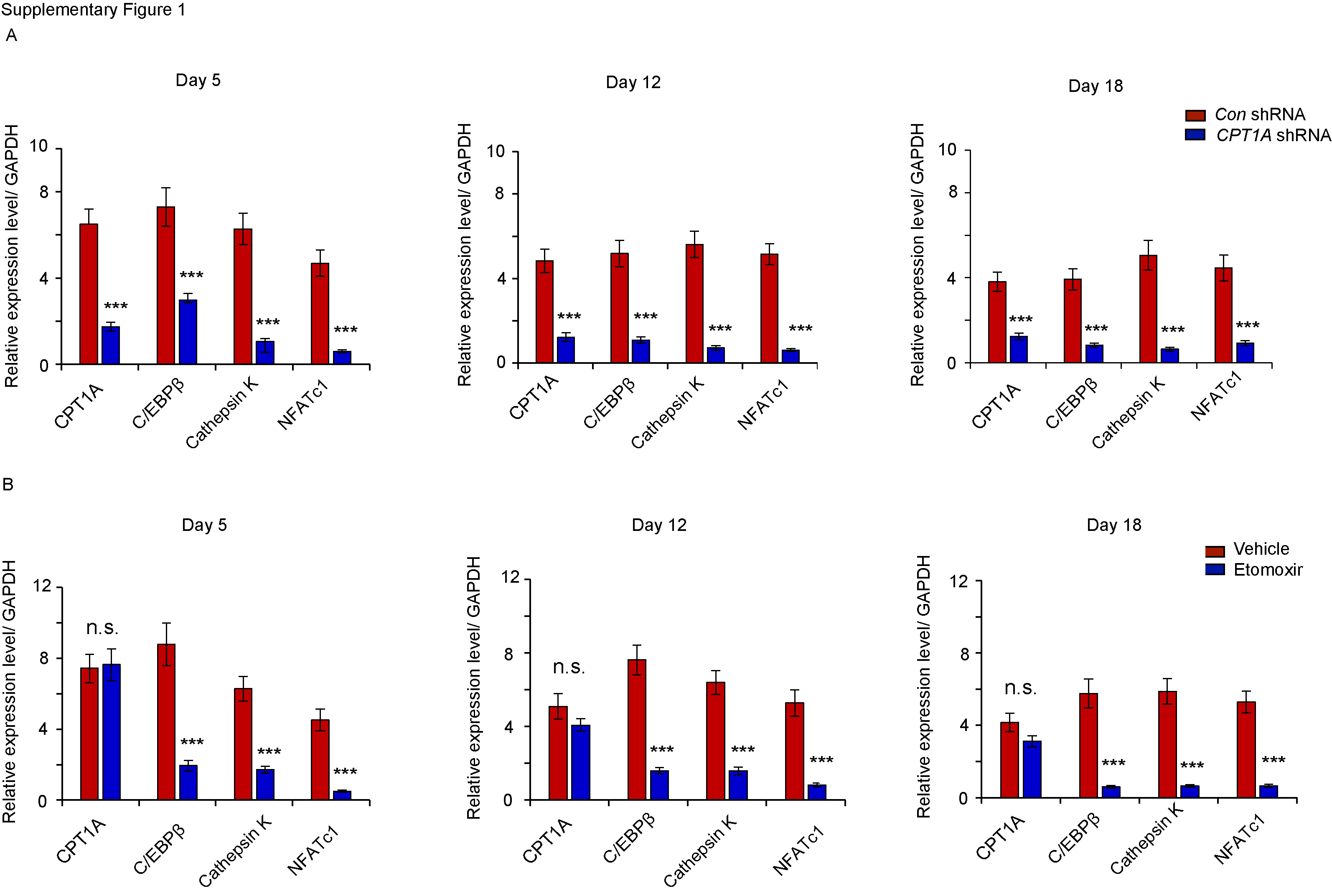

Supplement: Supplementary Figure 1 — mRNA levels of CPT1A and osteoclast differentiation markers at different stages of OC development. (A) CPT1A shRNA. CD14+ monocytes from RA patients were transfected with short hairpin RNA expression lentivirus for CPT1A knockdown (pLV-mCherry-U6>CPT1A_shRNA) or with (pLV-mCherry-U6>Scramble_ shRNA) as a control. Transcript levels of CPT1A, C/EBPβ, Cathepsin K, and NFATc1 were quantified by qPCR on day 5, day 12, and day 18 during osteoclast differentiation. (B) CPT1A inhibitor. RA-derived monocytes were treated with control or 25 μM etomoxir after M-CSF and RANKL stimulation. Transcript levels of CPT1A, C/EBPβ, Cathepsin K, and NFATc1 were quantified by qPCR on day 5, day 12, and day 18 during osteoclast differentiation. [file Image_1.tif]
